# Supplementary material for: The Burden of Spinal Muscular Atrophy on Informal Caregivers
Source: Int J Environ Res Public Health. 2020 Dec 2;17(23):8989. doi: 10.3390/ijerph17238989 (PMC7730048; doi:10.3390/ijerph17238989)
Supplement: Supplementary file 1 [file ijerph-17-08989-s001.pdf]

S1 Table: Mean hours (SD) of informal caregiving provided daily to patients with SMA by age.

Activities	<10 years old (n=46)		>10years old (n=22)	
	Main caregiver	All caregivers	Main caregiver	All caregivers
Basic hygiene, dressing.	1.11 (0.98)	1.65 (1.53)	1.37 (1.14)	1.86 (1.37)
Feeding the patient.	1.35 (1.64)	1.83 (2.25)	0.82 (0.76)	1.14 (1.05)
Bathing or showering	0.53 (0.34)	0.78 (0.57)	0.91 (0.81)	1.54 (1.35)
Helping the patient to move.	1.84 (2.28)	2.54 (2.82)	1.34 (1.53)	1.89 (1.78)
Total BADL	4.84 (3.49)	6.83 (4.79)	4.45 (3.71)	6.44 (4.84)
Cooking and preparing special meals.	0.55 (0.74)	0.85 (1.11)	0.6 (0.77)	0.9 (1.11)
Administering drugs/minor cures.	0.92 (1.16)	1.27 (1.61)	1.12 (1.18)	1.45 (1.31)
Other IADL[a]	0.02 (0.13)	0.04 (0.28)	0.01 (0.07)	0.01 (0.07)
Total IADL	1.5 (1.53)	2.16 (2.25)	1.75 (1.45)	2.37 (1.97)
Other activities directly related to the disease	0.51 (0.98)	0.72 (1.15)	0.73 (0.91)	1.81 (2.1)
All activities	6.86 (4.43)	9.72 (6.26)	6.94 (5.08)	10.64 (7.58)

Note: duration of informal care was censored to a maximum of 16 hours per day per caregiver. [a] Other IADL included the time spent on medical visits, diagnostic tests, travel, financial, administrative or legal affairs and social and leisure activities.

S2 Table: Mean hours (SD) of informal caregiving provided daily to patients with SMA by non-invasive respiratory supply system.

Activities	No (n=35)		Yes (n=30)	
	Main caregiver	All caregivers	Main caregiver	All caregivers
Basic hygiene, dressing	0.84 (0.74)	1.23 (1.4)	1.61 (1.15)	2.23 (1.18)
Feeding the patient	0.88 (0.95)	1.1 (1.16)	1.62 (1.8)	2.32 (2.52)
Bathing or showering	0.47 (0.42)	0.77 (0.89)	0.88 (0.63)	1.33 (0.95)
Helping the patient to move	1.65 (2.29)	2.31 (3.04)	1.86 (1.88)	2.48 (1.94)
Total BADL	3.86 (3.45)	5.42 (4.85)	5.98 (3.36)	8.38 (4.15)
Cooking and preparing special meals	0.42 (0.57)	0.61 (0.92)	0.79 (0.88)	1.24 (1.23)
Administering drugs/minor cures	0.85 (1.33)	1.05 (1.5)	1.12 (0.92)	1.57 (1.37)
Other IADL[a]	0.02 (0.15)	0.05 (0.33)	0.01 (0.06)	0.01 (0.06)
Total IADL	1.3 (1.42)	1.72 (1.93)	1.93 (1.56)	2.83 (2.26)
Other activities directly related to the disease	0.54 (0.81)	1.02 (1.38)	0.66 (1.15)	1.18 (1.86)
All activities	5.71 (4.26)	8.17 (6.25)	8.59 (4.61)	12.39 (6.44)

Note: duration of informal care was censored to a maximum of 16 hours per day per caregiver. [a] Other IADL included the time spent on medical visits, diagnostic tests, travel, financial, administrative or legal affairs and social and leisure activities.

S3 Table : Mean hours (SD) of informal caregiving provided daily to patients with SMA by country.

Activities	Spain		United Kingdom		Germany		France		Whole sample	
	Main caregiver	All caregivers	Main caregiver	All caregivers	Main caregiver	All caregivers	Main caregiver	All caregivers	Main caregiver	All caregivers
-Basic hygiene, dressing.	1.10 (1.21)	1.57 (1.7)	1.07 (0.71)	1.70 (1.13)	1.21 (0.86)	1.73 (1.04)	1.44 (1.11)	2.01 (1.68)	1.20 (1.04)	1.73 (1.48)
-Feeding the patient.	0.91 (0.94)	1.15 (1.22)	2.50 (2.73)	3.55 (3.69)	0.86 (0.69)	1.24 (0.83)	1.02 (0.86)	1.40 (1.34)	1.18 (1.43)	1.61 (1.97)
-Bathing or showering	0.54 (0.51)	0.85 (0.83)	0.53 (0.25)	0.96 (0.65)	0.83 (0.53)	1.15 (0.76)	0.78 (0.77)	1.28 (1.42)	0.66 (0.56)	1.03 (0.96)
-Helping the patient to move.	1.59 (2.17)	2.22 (4.48)	1.80 (2.31)	2.32 (2.23)	1.93 (1.77)	2.66 (2.19)	1.53 (2.17)	2.27 (2.90)	1.68 (2.08)	2.34 (2.54)
-Total BADL	4.15 (3.35)	5.79 (4.48)	5.89 (4.37)	8.52 (5.56)	4.84 (3.41)	6.77 (3.68)	4.76 (3.49)	6.95 (5.57)	4.72 (3.54)	6.71 (4.78)
-Cooking and preparing special meals.	0.55 (0.77)	0.89 (1.26)	0.61 (0.90)	0.82 (1.06)	0.89 (0.56)	1.36 (0.63)	0.31 (0.70)	0.44 (1.09)	0.57 (0.75)	0.87 (1.10)
-Administering drugs/minor cures.	1.10 (1.31)	1.35 (1.54)	1.39 (1.46)	1.95 (1.92)	0.77 (0.76)	1.13 (0.93)	0.73 (0.97)	1.06 (1.61)	0.99 (1.17)	1.33 (1.52)
-Other IADL[a]	0.03 (0.18)	0.07 (0.38)	0.00 (0.01)	0.00 (0.01)	0.02 (0.09)	0.02 (0.09)	0.00 (0.00)	0.00 (0.00)	0.02 (0.12)	0.03 (0.24)
-Total IADL	1.69 (1.64)	2.32 (2.33)	2.00 (1.67)	2.77 (2.22)	1.68 (1.13)	2.51 (1.43)	1.05 (1.42)	1.49 (2.34)	1.59 (1.50)	2.24 (2.16)
-Other activities directly related to the disease.	0.69 (1.17)	0.99 (1.40)	0.52 (1.02)	1.21 (1.87)	0.70 (0.47)	1.38 (1.24)	0.38 (0.89)	0.88 (2.03)	0.59 (0.96)	1.08 (1.59)
-All activities	6.52 (4.74)	9.10 (6.41)	8.42 (4.67)	12.50 (5.96)	7.21 (4.17)	10.65 (5.45)	6.18 (4.91)	9.32 (8.44)	6.89 (4.62)	10.02 (6.68)

Note: time of informal care was censored to a maximum of 16 hours per day per caregiver. [a] Other IADL included the time spent on medical visits, diagnostic tests, travelling, financial, administrative or legal affairs and social and leisure activities.

Table S4: Analysis of burden of care according to Zarit Burden Interview results

	Beta Coef. (Standard Error)	p-value
Type 1	2.73 (9.48)	0.78
Type 2	10.30 (6.32)	0.111
n		52
F		3.47
R square		0.41

Note: regression models included as control variables sex, age, country, marital and job status of caregivers and whether the patient with SMA was receiving social services due to the illness. a These values represent the beta coefficients obtained with ordinary least squares regression (OLS) robust of heteroscedasticity.
